# Absolute quantification revealed that glutamate increased the abundance of the rhizosphere bacterial community in Camellia oil tree under drought stress

**DOI:** 10.3389/fmicb.2025.1598000

**Published:** 2025-06-13

**Authors:** Kaizheng Lu, Junqin Zhou, Jun Yuan, Jiaqi Qiu, Xiaofeng Tan

**Affiliations:** ^1^Key Laboratory of Utilization of Woody Oil Resource, Central South University of Forestry and Technology, Changsha, China; ^2^Yuelu Mountain Laboratory, Central South University of Forestryand Technology, Changsha, China; ^3^Key Laboratory of Cultivation and Protection for Non-Wood Forest Trees, Ministry of Education, Central South University of Forestry and Technology, Changsha, China; ^4^College of Landscape Architecture, Central South University of Forestry and Technology, Changsha China; ^5^Weichang Manzu Mongol Autonomous County Forestry and Grassland Administration, Chengde, China

**Keywords:** glutamate, drought, Camellia oil tree, absolute quantification, rhizosphere, bacteria

## Abstract

**Introduction:**

Seasonal drought associated with the subtropical monsoon climate significantly impairs the growth and development of Camellia oil tree seedlings. While previous studies have established that drought stress elevates glutamate content in the rhizosphere of Camellia oil tree, the mechanisms through which glutamate modulates rhizosphere microbial community assembly remain unresolved.

**Methods:**

To investigate the effects of glutamate on the rhizosphere environment under drought stress, we conducted an experiment using three-year-old potted seedlings subjected to moderate drought. These seedlings were irrigated with 50 mL of glutamate solutions at varying concentrations (0, 1, 2, 5, and 10 mmol/L; labeled G0, G1, G2, G5, and G10, respectively). Through analysis of rhizosphere soil nutrients, enzyme activity, and bacterial community abundance (relative and absolute).

**Results:**

The study revealed the following: Concentrations of available nitrogen forms (DON, NH_4_^+^-N, NO_3_^–^-N) increased proportionally with glutamate concentration, whereas soil pH and urease activity exhibited inverse trends. Alpha and beta diversity analyses demonstrated significant divergence in bacterial community composition across treatments. Kruskal-Wallis, ANOVA, and LEfSe analyses identified 24 bacterial phyla significantly associated with treatment differences, with their abundance patterns corresponding to nitrogen cycling gene dynamics—generally peaking at G5 before declining.

**Discussion:**

These findings collectively suggest that 5 mmol/L Glu represents a pivotal concentration influencing rhizosphere bacterial community dynamics in Camellia oil tree under drought stress.

## Introduction

Camellia oil tree, a perennial evergreen shrub or small tree indigenous to China, is a vital woody oilseed species. Its seed-derived oil is widely recognized as a premium edible oil. Crucially, this species predominantly thrives in hilly regions, effectively alleviating arable land pressure and mitigating competition with staple crops, thereby contributing to national food security. The escalating impacts of global climate change have established drought as a critical constraint on Camellia oil tree growth and productivity (Tan, 2023; Zhou et al., 2023). Previous studies showed that glutamate content in rhizosphere soil was closely related to the degree of drought (Yuan et al., 2024). Glutamate (Glu), a multifunctional metabolic product, serves as a critical amino acid in plant systems. As a central component of nitrogen metabolism, Glu and its derivatives directly participate in synthesizing and metabolizing nitrogen-containing compounds, including carbohydrates, fatty acids, and other amino acids, thereby bridging carbon and nitrogen metabolic pathways (Liao et al., 2022). Over 95% of plant NH_4_^+^—derived from root absorption or nitrate reductase (NR)- and nitrite reductase (NiR)-mediated NO_3_^–^ reduction—is assimilated via the glutamine synthetase/glutamate synthase (GS/GOGAT) cycle, where Glu acts as a pivotal intermediary (Fortunato et al., 2023). This underscores its indispensable role in nitrogen metabolism in higher plants.

Amino acids, including Glu, are increasingly incorporated into organic fertilizers to mitigate abiotic stresses. Functioning as a primary metabolite, nitrogen source, and signaling molecule, Glu contributes to plant adaptation under stress conditions (Liao et al., 2022; Qiu et al., 2020). For instance, foliar application of 4 mmol/L Glu under drought stress enhances wheat dry matter accumulation, photosynthetic efficiency, and grain quality while minimizing yield loss (Li et al., 2024). When utilized as a nitrogen source in rice, Glu is rapidly metabolized into compounds like glutamine rather than accumulating (Kan et al., 2017). Furthermore, Glu bolsters plant tolerance to cold, salinity, cadmium, and arsenic by improving photosynthetic capacity and antioxidant system performance (Asgher et al., 2022; Franzoni et al., 2022; Gai et al., 2020; Lee et al., 2021). However, excessive Glu accumulation under NH_4_^+^ stress can impair the tricarboxylic acid (TCA) cycle, suppressing plant growth (Wang et al., 2020). Beyond stress adaptation, Glu influences microbial community dynamics. In strawberry anthosphere studies, Glu supplementation drastically altered microbial composition, with Streptomycetaceae dominating 99.9% of the community by weeks 6 and 8 post-treatment (Kim et al., 2021). Amino acids, as vital nitrogen sources for plants and microbes, trigger competitive uptake in soil systems: microbes rapidly assimilate these compounds, though plants exhibit superior absorption efficiency (Jones et al., 2005). Similarly, organic matter enriched with phenylalanine or leucine enhances humus formation via bacterial activity (Zheng et al., 2021).

While Glu research has predominantly focused on plant growth regulation, shifts in rhizosphere microbiomes remain underexplored. This gap is particularly significant given that root-associated microbial communities— often termed the plant second genome, play essential roles in supporting plant growth and development through multiple mechanisms (Berendsen et al., 2012). Advancing our understanding of how rhizosphere microbes mediate plant adaptation to abiotic stress is pivotal for enhancing future resilience to extreme environmental conditions.

Current analytical methodologies for assessing microbial dynamics are constrained by critical limitations. Although relative quantification sequencing has been extensively employed to link shifts in taxonomic composition with environmental gradients (Props et al., 2017; Wang et al., 2024), this approach yields a partial understanding of microbial ecology. Emerging evidence demonstrates that apparent increases in relative abundance of specific taxa might represent not biological proliferation but rather the competitive suppression of coexisting species. Conversely, absolute quantification sequencing enables precise detection of subtle microbial population fluctuations at heightened resolution, thereby providing superior capacity to monitor community-level adaptations to environmental stressors (Maghini et al., 2024; Tkacz et al., 2018). This methodological dichotomy highlights the imperative for incorporating absolute quantification frameworks to resolve complex plant-microbe interaction mechanisms under abiotic stress conditions.

Previous studies by our group have demonstrated that Glu content in Camellia oil tree rhizosphere soil rises with escalating drought severity. However, whether this drought-induced Glu accumulation induces structural shifts in the rhizosphere microbiome remains unresolved. In this study, we integrate absolute and relative quantification data to analyze the effects of exogenous Glu on the rhizosphere microbial community of Camellia oil tree, thereby elucidating its regulatory mechanisms on microbial composition and function. These findings aim to establish a theoretical foundation for development and utilization of rhizosphere microorganisms in the future.

## 2 Materials and methods

### 2.1 Experiment design

The experiment was conducted at the nursery of Central South University of Forestry and Technology, situated in Changsha, Hunan Province, China (28°11’N, 113°04’E), which features a subtropical monsoon climate with a mean annual precipitation of 1,361 mm and an average temperature of 17.2°C. In late November 2023, 3-year-old Camellia oil tree “Huashuo” seedlings were transplanted into plastic pots (30 cm height × 25 cm diameter) filled with lateritic soil collected from a Camellia oil tree plantation. Prior to transplantation, the soil was manually cleared of plant debris and sieved through a 2-mm mesh. After an 8-month pre-cultivation phase involving periodic pruning to standardize plant growth, drought pre-treatment commenced on 20 June 2024. The pre-treatment protocol comprised initial saturation watering (until drainage occurred from pot bases) followed by complete cessation of irrigation. Soil water content (SWC) was monitored daily via gravimetric methods. The experimental drought treatment was initiated when SWC declined to 20–25% (m/m), corresponding to moderate drought thresholds as per national agricultural meteorological standards (National Technical Committee for Agricultural Meteorology Standardization, 2015). All measurements and procedures began immediately upon attaining this target SWC level.

The experiment commenced on 1 July 2024 with five treatments: ① Root application of 50 mL deionized water (G0); ② Root application of 50 mL 1 mmol/L Glu solution (G1); ③ Root application of 50 mL 2 mmol/L Glu solution (G2); ④ Root application of 50 mL 5 mmol/L Glu solution (G5); ⑤ Root application of 50 mL 10 mmol/L Glu solution (G10). The experimental design adopted a randomized block arrangement with three blocks per treatment group, each containing five replicates (*n* = 15 per treatment). Glu was applied only once; during subsequent treatments, only watering was conducted to maintain soil moisture and weeds were regularly removed. Glu (≥ 98.5% purity; Hushi Co., China) was dissolved in deionized water to prepare the designated concentrations. Soil moisture was maintained at 20–25% (m/m) through daily gravimetric monitoring. Pots were weighed daily at 08:00, and weight loss attributable to evapotranspiration was offset by supplemental irrigation to restore the target soil moisture level. This protocol ensured consistent drought stress across all treatments throughout the experimental period.

### 2.2 Sample collection

Following 30 days of treatment, Camellia oil tree seedlings were carefully excavated. Root systems were gently cleaned through manual removal of loosely adhered soil aggregates, followed by meticulous brushing to dislodge rhizosphere soil particles. The collected soil was homogenized using a 2-mm sieve and divided into two aliquots. Cryopreserved subsamples were flash-frozen in liquid nitrogen and stored at -80°C in an ultra-low temperature freezer for subsequent 16S rRNA sequencing-based analysis of microbial communities. The remaining soil samples were air-dried and used to determine physicochemical properties.

#### 2.2.1 Soil nutrient and enzyme activity measurement

Soil pH was measured using a pH meter (Sartorius, Germany) with a soil-to-water ratio of 1:5. Soil organic carbon (SOC) was determined using the potassium dichromate-sulfuric acid colorimetric method. Total nitrogen (TN) content was measured using the Kjeldahl method (Kirk, 2002). Ammonium nitrogen (NH_4_^+^-N) was determined using the indophenol blue colorimetric method. Nitrate nitrogen (NO_3_^–^-N) was extracted with potassium chloride solution and analyzed using a discrete chemical analyzer (SmartChem 200). Dissolved organic nitrogen (DON) was extracted from soil using deionized water and also measured with the discrete chemical analyzer (Jones and Willett, 2006). Total phosphorus (TP) and available phosphorus (AP) were determined using the sodium hydroxide fusion-molybdenum antimony colorimetric method and the Mehlich 3 method, respectively (Daniels et al., 2001). Total potassium (TK) and available potassium (AK) were analyzed using a flame photometer (Gao et al., 2019).

Catalase (CAT) activity was determined using a titration method (Goldblith and Proctor, 1950), while urease (Ure) activity was measured using the phenol-sodium hypochlorite colorimetric method, with one unit of enzyme activity defined as the amount of enzyme that produces 1 mg of NH_4_^+^-N per gram of soil within 24 h (Kandeler and Gerber, 1988). Phytase (Phy) activity was assessed by hydrolyzing sodium phytate to release inorganic phosphorus, which reacts with molybdate color reagent under acidic conditions to form a blue complex, and absorbance was measured at 700 nm. One unit of Phy activity was defined as the release of 1 μMol of inorganic phosphorus per gram of soil per hour (Jackman and Black, 1952). The activities of acid phosphatase (ACP), β-glucosidase (βG), and leucine aminopeptidase (LAP) were measured using the p-nitrophenol (pNP)-based microplate method.

#### 2.2.2 Method for absolute quantification of 16S rRNA amplicon sequencing

Total genomic DNA was extracted using the FastDNA SPIN Kit for Soil (MP Biomedicals, Santa Ana, CA) according to the manufacturer’s instructions. The integrity of genomic DNA was detected through agarose gel electrophoresis, and the concentration and purity of genomic DNA were detected through the Nanodrop 2000 and Qubit3.0 Spectrophotometer. Multiple spike-ins with identical conserved regions to natural 16S rRNA genes and variable regions replaced by random sequence with ∼40% GC content were artificially synthesized. Then, appropriate proportion of spike-ins mixture with known gradient copy numbers were added to the sample DNA. The V3-V4 hypervariable regions of the 16S rRNA gene and spike-ins were amplified with the primers 341F (5-CCTACGGGNGGCWGCAG-3) and 805R (5-GACTACHVGGGTATCTAATCC-3) and then sequenced using Illumina NovaSeq 6000 sequencer.

The raw read sequences were processed in QIIME2 (Bolyen et al., 2019). The adaptor and primer sequences were trimmed using the cutadapt plugin. DADA2 plugin was used for quality control and to identify amplicon sequence variants (ASVs) (Callahan et al., 2016). Taxonomic assignments of ASV representative sequences were performed with confidence threshold 0.7 by a pre-trained Naive Bayes classifier which was trained on the SILVA (version 138.2). Then the spike-in sequences were identified, and reads were counted. Standard curve for each sample was generated based the read-counts versus spike-in copy number, and the absolute copy number of each ASV in each sample was calculated by using the read-counts of the corresponding ASV. Since the spike-in sequence is not a component of the sample flora, the spike-in sequence needs to be removed in the subsequent analysis (Jiang et al., 2019).

### 2.3 Data analysis

Data organization was performed using Microsoft 365. One-way ANOVA was conducted in SPSS 22.0 (IBM, USA) with Duncan’s *post-hoc* test to determine significant differences (significance threshold, *P* < 0.05). All statistical analyses were implemented in R (v4.3.2) using standardized bioinformatics workflows. Alpha-diversity index were calculated with the vegan and ade4 packages, followed by Principal Component Analysis (PCA) to evaluate β-diversity patterns, validated through permutational multivariate analysis of variance (PERMANOVA; 999 permutations). Hypothesis testing included one-way ANOVA with Tukey HSD *post hoc* comparisons, Wilcoxon rank-sum tests, and Kruskal-Wallis tests. Temporal trends of differentially abundant taxa were analyzed via fuzzy c-means clustering (Mfuzz), while functional predictions were generated using PICRUSt2 with KEGG Orthology annotations. All visualizations (boxplots, bar charts, Venn diagrams, heatmaps) were produced in ggplot2 following data-ink optimization principles (Dixon, 2003; Dray and Dufour, 2007; Kumar and Futschik, 2007; Wickham, 2016). A partial least squares path model (PLS-PM) was conducted to determine the direct and indirect effects of Glu content and microbial communities using the “plspm” package in R (version 4.4.3). Prior to this step, a collinearity analysis was performed on all indicators, and those with a Variance Inflation Factor (VIF) greater than 10 were excluded. In the partial least squares (PLS) analysis, latent variables were defined as: Glu concentrations (0, 1, 2, 5, 10 mmol/L), soil nutrients (sub-variables: pH, SOC, TK, AP, DON), soil enzymes (sub-variables: Phy, BG), and alpha diversity (sub-variables: Shannon). The process of the construction of the model reference.^1^ After standardizing the data, the metric PLS method was applied using the centroid weighting scheme. The algorithm was run for a maximum of five iterations and terminated when the convergence tolerance reached 1 × 10^–6^. The overall model fit was appropriately classified into weak, moderate, and strong according to threshold values of 0.1, 0.25, and 0.36 for the goodness-of-fit (GoF) index (Wetzels et al., 2009). Finally, we calculated the GoF for component-based and covariance-based PLS-PM.

## 3 Results

### 3.1 Effect of different glutamate concentrations on soil physicochemical properties and enzyme activities

The application of different Glu concentrations induced significant variations in soil physicochemical properties (Table 1). Soil pH declined progressively with increasing Glu concentration, reaching its lowest value (4.66) in the G10 treatment, though no significant difference was observed compared to G5. SOC, TN, TP, TK, AP, and AK contents peaked under the G5 treatment, significantly exceeding those in G0, G1, and G2. In contrast, DON, NH_4_^+^-N, and NO_3_^–^-N levels attained their maxima under G10, with values of 72.02, 13.47, and 54.08 mg/kg, respectively.

**TABLE 1:** Soil physicochemical characteristics by treatments.

Treatments	Ph g/kg	SOC g/kg	TN g/kg	TP g/kg	TK g/kg	AP mg/kg	AK mg/kg	DON mg/kg	NH_4_^+^-N mg/kg	NO_3_^–^-N mg/kg
G0	4.82 ± 0.03b	45.78 ± 0.34b	2.27 ± 0.06cd	1.99 ± 0.18b	10.55 ± 0.18b	59.35 ± 10.67bc	155.13 ± 6.38c	26.19 ± 2.57c	6.69 ± 0.57b	46.52 ± 0.25d
G1	5.16 ± 0.05a	50.22 ± 0.79b	2.48 ± 0.20bc	2.09 ± 0.32b	11.08 ± 0.76ab	65.77 ± 13.63b	169.68 ± 6.84b	36.96 ± 8.90b	12.37 ± 1.57a	52.42 ± 0.57b
G2	4.91 ± 0.02b	46.61 ± 6.31b	2.19 ± 0.06d	1.45 ± 0.17c	10.58 ± 0.09b	46.08 ± 1.66c	154.00 ± 2.23c	24.92 ± 1.74c	6.77 ± 0.26b	36.28 ± 0.47e
G5	4.79 ± 0.15bc	79.71 ± 18.81a	2.85 ± 0.14a	2.67 ± 0.30a	11.46 ± 0.35a	91.70 ± 1.91a	208.28 ± 8.27a	39.93 ± 3.00b	7.53 ± 0.30b	48.30 ± 1.14c
G10	4.66 ± 0.01c	62.56 ± 7.33ab	2.70 ± 0.11ab	2.41 ± 0.09ab	11.47 ± 0.50a	97.58 ± 5.10a	155.53 ± 1.63c	72.02 ± 4.05a	13.47 ± 0.54a	54.08 ± 1.11a

G0, G1, G2, G5, and G10 represent root treatments with Glu solutions of 0, 1, 2, 5, and 10 mmol/L, respectively. Different lowercase letters indicate significant differences between treatments (*P* < 0.05).

Rhizosphere enzyme activities exhibited Glu concentration-dependent trends (Table 2). Maximal CAT, Phy, acid ACP, and βG activities were recorded under G10 at 2.68 mL/g, 0.63 μmol/g/h, 271.95 nmol/g/h, and 60.41 nmol/g/h, respectively. These values were significantly elevated relative to G0, corresponding to 1. 5-, 1. 4-, 2. 2-, and 2.4-fold increases. Conversely, Ure and LAP activities showed a general decline with rising Glu concentrations, reaching 0.31 mg/g and 3.84 nmol/g/h under G10. No significant differences in Phy, ACP, or LAP activities were detected between G10 and G5.

**TABLE 2:** Soil enzyme activities by treatments.

Treatments	CAT ml/g	Ure mg/g	Phy μmol/g/h	ACP nmol/g/h	βG nmol/g/h	LAP nmol/g/h
G0	1.74 ± 0.14c	0.46 ± 0.01a	0.44 ± 0.02b	126.11 ± 15.66b	25.19 ± 1.55c	2.49 ± 0.02d
G1	2.34 ± 0.37ab	0.42 ± 0.02b	0.44 ± 0.14b	283.87 ± 14.35a	57.78 ± 0.49a	5.02 ± 0.02a
G2	2.21 ± 0.29b	0.39 ± 0.01c	0.51 ± 0.04ab	276.28 ± 25.41a	60.52 ± 5.58a	4.74 ± 0.12b
G5	1.77 ± 0.06c	0.38 ± 0.01c	0.59 ± 0.05ab	260.08 ± 8.71a	50.13 ± 3.63b	3.84 ± 0.04c
G10	2.68 ± 0.13a	0.31 ± 0.02d	0.63 ± 0.12a	271.95 ± 8.04a	60.41 ± 4.62a	3.84 ± 0.12c

G0, G1, G2, G5, and G10 represent root treatments with Glu solutions of 0, 1, 2, 5, and 10 mmol/L, respectively. Different lowercase letters indicate significant differences between treatments (*P* < 0.05).

### 3.2 Glutamate increased the alpha diversity of the bacterial community

**FIGURE 1:**
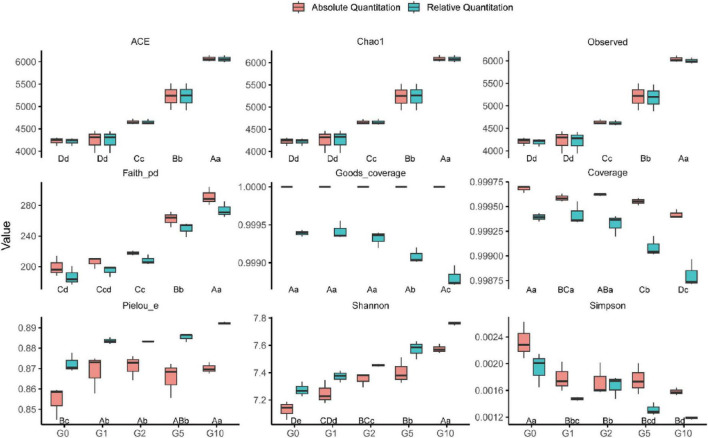
Bacterial community alpha diversity under different treatments. Uppercase letters denote statistical differences between absolute quantification treatments (*P* < 0.05); lowercase letters indicate differences between relative quantification treatments (*P* < 0.05).

The alpha diversity index of bacterial communities, including both relative and absolute abundance-based measures, exhibited consistent trends across treatments (Figure 1). Species richness index (ACE, Chao1, and Observed) increased with rising Glu concentrations, peaking in G10 and reaching the lowest values in G0, though no significant difference occurred between G0 and G1. Faith’s phylogenetic diversity (Faith_pd) mirrored this richness pattern, with maximal values in G10. Absolute abundance-based measures revealed no significant differences among G0, G1, and G2, whereas relative abundance analysis detected a significant divergence between G0 and G2. Coverage index showed no significant variation among G0, G1, and G2; however, under relative quantification, these treatments exhibited significantly higher coverage than G5 and G10.

Pielou’s evenness (Pielou_e) under relative quantification was significantly elevated in G10 compared to other treatments, with G1, G2, and G5 showing no significant differences but all exceeding G0. Conversely, absolute quantification demonstrated significantly lower Pielou_e in G0 relative to Glu-treated groups, which showed no intergroup differences. The Shannon index increased progressively with Glu concentration under both quantification methods, attaining maximal values in G10. In contrast, the Simpson index displayed an inverse trend, peaking in G0 and reaching its nadir in G10.

### 3.3 Treatment-dependent divergence in bacterial β-diversity

**FIGURE 2:**
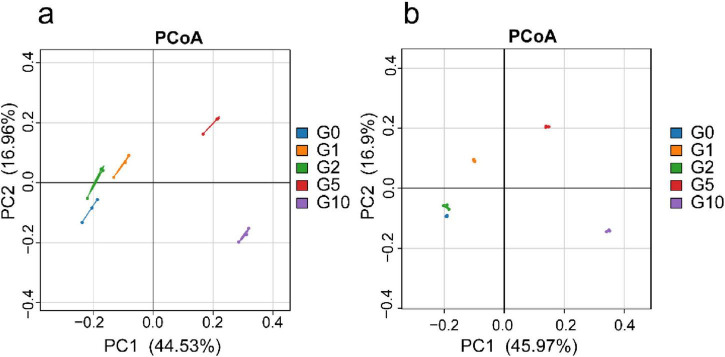
Bacterial β-diversity under different treatments. **(a)** Depicts Principal Coordinate Analysis (PCoA) of treatment groups based on absolute quantification; **(b)** Illustrates PCoA under relative quantification. The straight lines between samples represent connectivity links; they do not possess statistical significance.

Principal Coordinate Analysis (PCoA) revealed consistent overall patterns between absolute and relative quantification (Figure 2). The G5 and G10 treatments occupied the first and fourth quadrants, respectively, whereas G0, G1, and G2 clustered in the second and third quadrants, demonstrating distinct differentiation among treatments. Under absolute quantification, the first principal coordinate (PCoA1) accounted for 44.53% of variance, and the second axis (PCoA2) explained 16.96%. Similarly, relative quantification showed PCoA1 explaining 45.97% of variance and PCoA2 contributing 16.90%.

### 3.4 Glutamate increased the absolute abundance of bacterial phyla

**FIGURE 3:**
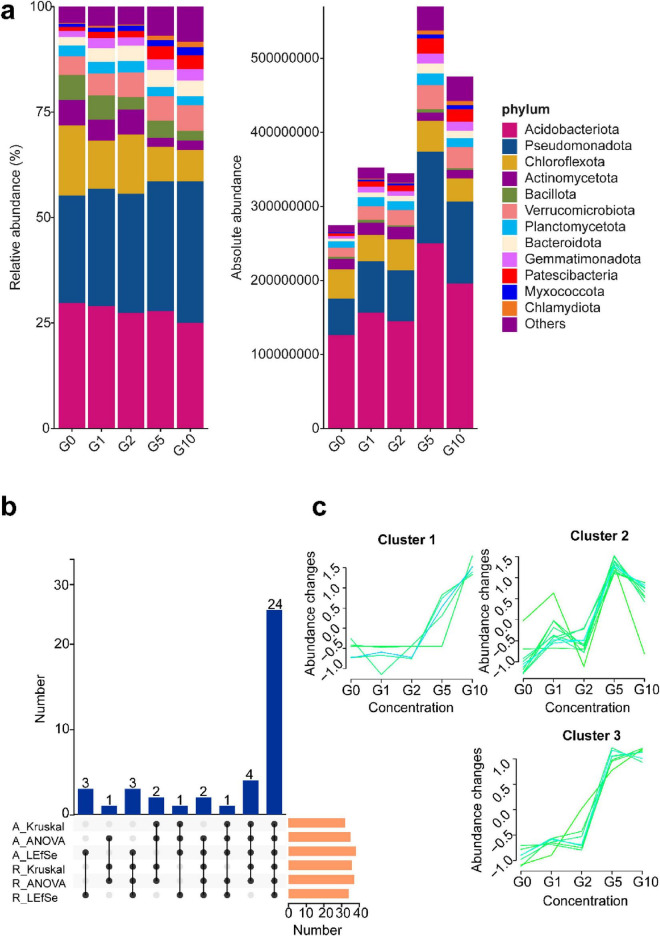
Community composition and differences under different treatments. (a) Left panel depicts bacterial community composition under relative quantification; right panel illustrates absolute quantification results, both analyzed at the phylum level. **(b)** Upset plot of differential phyla identified across absolute and relative quantification methods. **(c)** Temporal trend analysis of common differential phyla highlighted in **(b)**.

To assess the effects of varying Glu concentrations on rhizosphere soil community composition in Camellia oil tree, bar charts were generated for the top 12 phyla under both relative and absolute abundance metrics (Figure 3a). Marked discrepancies emerged between the two quantification methods. Under relative abundance, the collective proportions of Acidobacteriota, Chloroflexota, Actinomycetota, and Bacillota declined progressively with increasing Glu concentrations, while Pseudomonadota exhibited an inverse trend. Absolute abundance profiles, however, revealed divergent dynamics: Acidobacteriota and Pseudomonadota abundances increased with Glu concentration, peaking at the G5 treatment. This threshold concentration correlated with significantly elevated total bacterial abundance in G5 compared to other treatments.

To identify differential communities driving treatment variations, three analytical approaches—Kruskal-Wallis test, ANOVA, and LEfSe—were applied (Figure 3b). Absolute quantification detected 32, 35, and 38 differential communities via these methods, respectively; relative quantification identified 36, 37, and 34.

An upset plot visualized intersecting differential communities, revealing 24 taxa consistently identified across all methods (Figure 3c). These taxa clustered into three abundance trends: Cluster 1: Taxa in this group exhibited minimal changes under low Glu concentrations (G0–G2), followed by a sharp increase at G5 and G10. This suggests that these microbes may require a threshold level of Glu to activate growth or metabolic functions, potentially indicating specialization in amino acid assimilation under high nitrogen availability. Cluster 2: These taxa showed a complex abundance pattern, with peaks at G2 and G5 but a decline at G10. This fluctuation may reflect competitive dynamics or niche partitioning along the Glu gradient, suggesting that moderate concentrations may favor their activity, while excess nitrogen could inhibit or shift community interactions. Cluster 3 is similar with Cluster 2 but with attenuated responsiveness to low-concentration stimuli (G0–G2), this functional group reached maximal abundance at G5 followed by progressive attenuation through G10. It is worth noting that across all clusters, a marked shift in abundance occurred between G5 and G10 treatments.

### 3.5 Correlation of differential phyla and soil nutrients and enzyme activities

**FIGURE 4:**
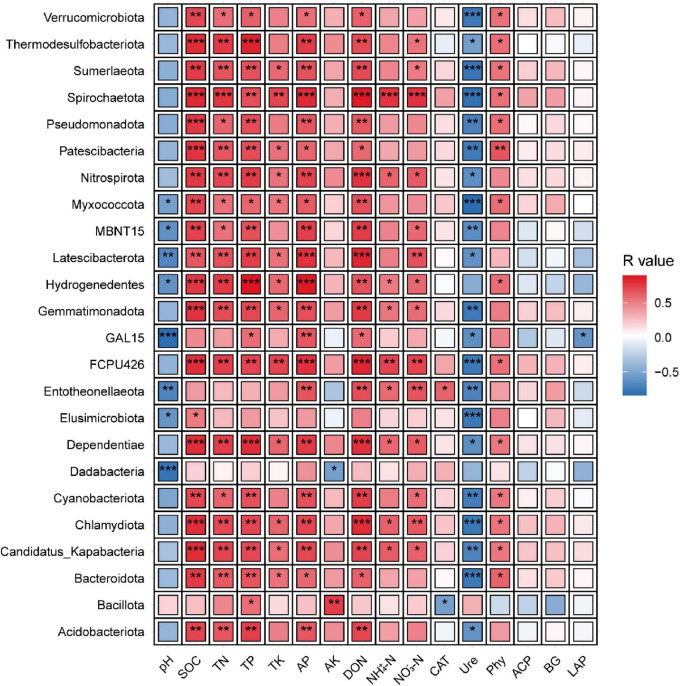
Correlation of differential communities with soil environmental factors. The phyla correspond to those exhibiting inter-treatment differences in Figure 3b. Color gradients denote correlation coefficients between phyla, where *indicates significance at *P* < 0.05, ** at *P* < 0.01, *** at *P* < 0.001.

Correlation analysis between the 24 identified phyla and soil nutrients/enzyme activities revealed that most phyla were strongly associated with soil N, and P dynamics (Figure 4). Specifically, 20 phyla (excluding GAL15, Entotheonellaeota, Dadabacteria, and Bacillota) exhibited significant positive correlations with SOC. Similarly, 19 phyla (excluding GAL15, Entotheonellaeota, Elusimicrobiota, Dadabacteria, and Bacillota) showed positive correlations with TN, while 21 phyla (excluding Entotheonellaeota, Elusimicrobiota, and Dadabacteria) were positively linked to TP. For AP and DON, 21 phyla (excluding Elusimicrobiota, Dadabacteria, and Bacillota) demonstrated significant positive associations. Conversely, Ure activity displayed an inverse pattern, with 21 phyla (excluding Hydrogenedentes, Dadabacteria, and Bacillota) showing significant negative correlations. These results underscore the prevalence of nutrient-driven phylum responses, contrasting sharply with the inhibitory relationship observed between phyla and Ure activity.

### 3.6 Glutamate promotes the nitrogen cycle

**FIGURE 5:**
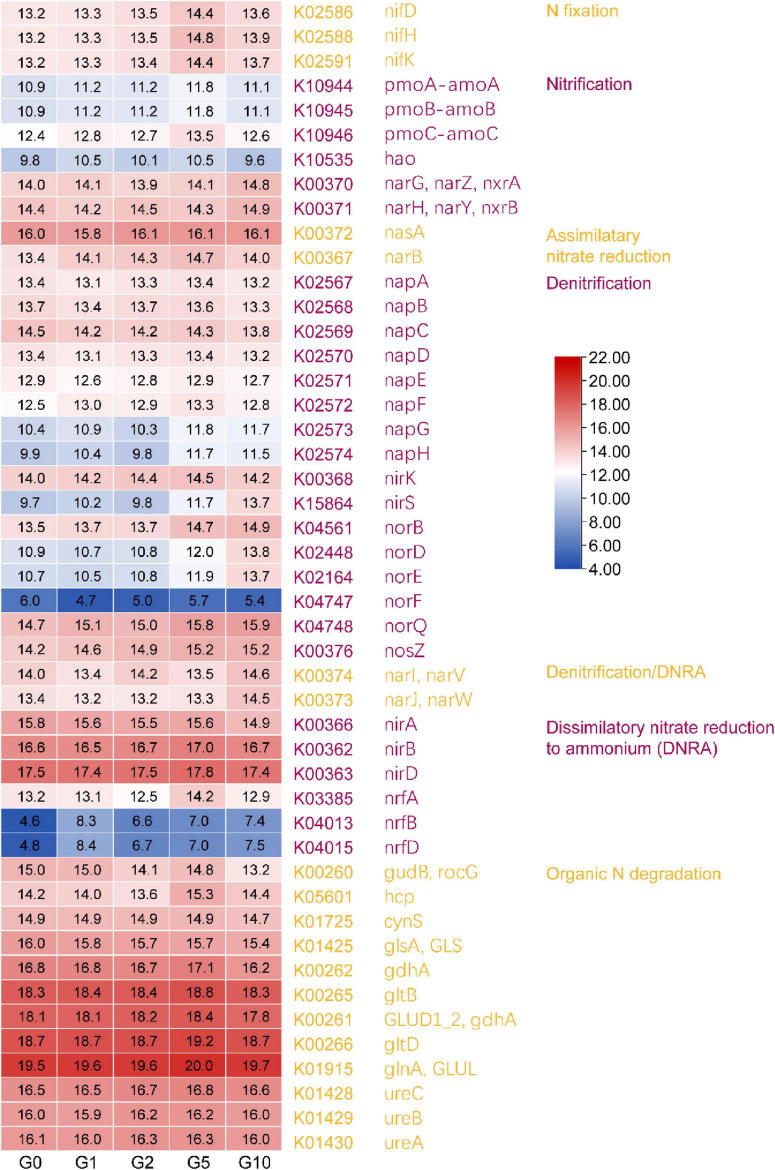
The differences in nitrogen-related genes of the differential communities predicted by PICRUST2. Values represented by different colors have been normalized (Log2 transformation).

To clarify the functional roles of the 24 differential phyla driving treatment variations, functional prediction analysis was conducted using PICRUSt2. Given Glu’s role as a nitrogen source, the investigation focused exclusively on nitrogen cycling gene abundance differences (Figure 5). Results revealed an overall trend of initial increase followed by decline in gene abundance. Genes associated with nitrogen fixation, nitrification (excluding hao), assimilatory nitrate reduction, denitrification (excluding *napB, napC, nirS, norB, norD, norE, norF*, and *norQ*), dissimilatory nitrate reduction to ammonium (excluding *nrfB* and *nrfD*), and organic nitrogen degradation (excluding *gudB* and *glsA*) all peaked in abundance under the G5 treatment.

### 3.7 The driving factors of bacteria diversity change

**FIGURE 6:**
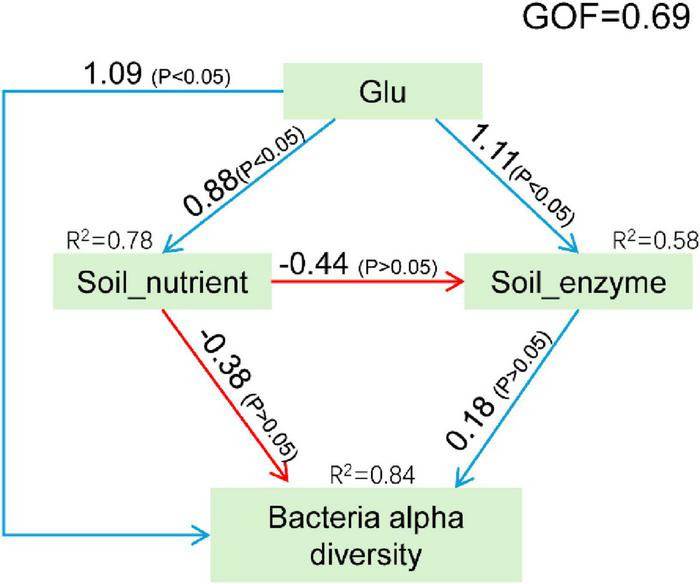
Structural equation model between environmental factors and bacterial diversity. The blue and red lines represent the positive and negative contributions between variables, respectively.

To more clearly elucidate the effects of Glu on rhizosphere soil microbial diversity, a structural equation model (SEM) was constructed (Figure 6). The PLS algorithm estimated path coefficients, demonstrating strong model explanatory power with a global GOF index of 0.69 (Akter et al., 2011). The results indicate that most manifest variables have been correctly assigned to their corresponding latent variable modules (Supplementary Tables 1,2). Notably, the latent variable representing pH is unique in exhibiting negative correlations with all associated explicit variables. Glu exerted the strongest positive effect on bacterial alpha diversity (coefficient = 1.09, *P* < 0.05), while soil nutrient levels showed the most pronounced negative effect (coefficient = -0.38, *P* > 0.05). Notably, Glu demonstrated a substantial positive influence on soil nutrient levels (coefficient = 0.88, *P* < 0.05). In contrast, soil enzyme activity exhibited only a minimal positive effect on bacterial alpha diversity (coefficient = 0.18, *P* > 0.05). Soil enzyme activity was also directly enhanced by Glu (coefficient = 1.11, *P* < 0.05) but negatively impacted by soil nutrient levels (coefficient = -0.44, *P* > 0.05).

Glu contributed 0.88, 0.73, and 0.89 to Soil_nutrient, Soil_enzyme, and Bacterial alpha diversity, respectively; while the contributions of Soil_nutrient and Soil_enzyme to Bacterial alpha diversity were -0.46 and 0.18, respectively (Supplementary Table 3).

In summary, bacterial alpha diversity was primarily influenced by Glu. Specifically, Glu concentration directly affected alpha diversity and indirectly modulated it through its impact on soil nutrient dynamics.

## 4 Discussion

This study demonstrated that exogenous Glu application under drought conditions predominantly altered nitrogen dynamics in the rhizosphere soil of Camellia oil tree, with DON being the most significantly impacted fraction. Bacterial community diversity trends were largely consistent between absolute and relative quantification methodologies. However, relative abundance quantification failed to reflect the pronounced inter-treatment disparities in Acidobacteriota and Pseudomonadota abundances. Functional prediction analysis further revealed that the observed inter-treatment differences were principally linked to nitrogen fixation, nitrification, and denitrification processes.

Discrepancies were observed between absolute and relative quantitative results for alpha and beta diversity. Relative quantification largely aligned with absolute quantification in treatments exhibiting pronounced differences, such as between G5 and G10 (Figures 2a,b). However, it inadequately resolved subtle differences between treatments with smaller variations, such as G0, G1, and G2. Additionally, relative quantification failed to capture changes in total bacterial community abundance under G5 and G10 treatments in analyses of community diversity (Figure 3a). These findings suggest that absolute quantification may be preferable when analyzing microbial datasets with minor inter-treatment differences.

This study demonstrated that increasing Glu application induced progressive soil acidification—a finding contrary to previous reports suggesting rhizosphere alkalization from exogenous arginine, proline, glutamine, or Glu due to ammonia accumulation (Liu et al., 2023). This discrepancy may stem from differences in rhizosphere microbiota between Camellia oil tree and model plants (e.g., Arabidopsis) or cultivation substrates (soil vs. artificial media). The acidic red soil used in this study, characteristic of subtropical monsoon regions, is non-calcareous with lower CaCO3 content compared to temperate calcareous soils. Consequently, its reduced acid-buffering capacity results in heightened sensitivity to nitrogen inputs and predisposition to acidification (Dong et al., 2025; Hao et al., 2022). Additionally, the molecular structure of Glu (two carboxyl groups and one amino group) may further exacerbate acidification through proton release during dissociation under acidic conditions (Bolan and Hedley, 2003). Despite decreasing pH trends, DON, NH_4_^+^-N, and NO_3_^–^-N concentrations increased proportionally with Glu dosage. As a plant-available nitrogen source, Glu supplementation through irrigation directly elevates soil nitrogen levels. However, TN content showed no significant variation between G5 and G10 treatments, potentially due to counterbalancing trends among other nitrogen pools—a phenomenon requiring further investigation.

Soil enzymes serve as critical biocatalysts in soil ecosystems, with their activity closely associated with nutrient availability (Aransiola et al., 2022). CAT and βG are centrally involved in the soil carbon cycle. CAT mitigates hydrogen peroxide accumulation, reducing its toxic effects on soil organisms while catalyzing biochemical reactions linked to microbial decomposition and metabolic processes. βG, a key component of the cellulase family, primarily facilitates the degradation of lignin and cellulose (Chen et al., 2022; Kou et al., 2022). Consistent with prior studies, CAT and βG activities decreased under low soil moisture but increased with exogenous nitrogen inputs (Bogati and Walczak, 2022; Dong et al., 2022). This phenomenon may be attributed to Glu’s role as a free amino acid, which is readily assimilated by soil microorganisms, enhancing nitrogen and carbon availability and stimulating microbial activity. Ure and LAP are integral to soil nitrogen dynamics: Ure regulates nitrogen availability through urea hydrolysis, while LAP releases leucine via protein hydrolysis (Chen et al., 2018). However, this study observed reduced Ure and LAP activities with increasing Glu concentrations, contrasting previous findings (Chen et al., 2018; Dong et al., 2022). This divergence likely stems from differences in nitrogen sources—high-purity Glu here versus mixed-form fertilizers in prior studies. Furthermore, direct rhizospheric application of dissolved Glu may have promoted preferential root uptake, limiting substrate availability for Ure and LAP and thereby suppressing enzymatic activity. Phy and ACP activities remained stable across treatments, likely due to the absence of phosphorus in Glu, precluding direct influence on soil phosphorus cycling.

To date, no studies have explicitly examined the effects of exogenous Glu on plant rhizosphere microbiota. This study observed increased Shannon and Chao1 index with escalating Glu dosage. SEM revealed that Glu exerted positive effects on both soil nutrients and bacterial alpha diversity, while soil nutrients demonstrated a negative regulatory effect on bacterial alpha diversity. A meta-analysis indicates that exogenous N application under field conditions reduces soil microbial Shannon and Chao1 index (Wang C. et al., 2018). This divergence may be attributable to three interrelated mechanisms: first, interference from co-occurring nitrogen sources. Prior meta-analyses predominantly derived data from fertilized agroecosystems or forests, where nitrogen inputs arise from anthropogenic activities or sustained organic matter decomposition (Wang C. et al., 2018). In this study, soil was sourced from an unfertilized Camellia oil tree plantation with surface litter removed, yielding nutrient-poor conditions. Consequently, limited nitrogen availability may have amplified microbial proliferation following Glu supplementation. Second, disparate research objectives: agricultural and forest studies typically prioritize yield enhancement and economic trait optimization through nitrogen addition, whereas this work focused on drought resilience—a distinction that likely influenced microbial community responses. Thirdly, the application of Glu concentrations significantly altered soil pH, which may negatively regulate bacterial alpha diversity through modified soil nutrient profiles. This finding suggest that Glu may play a dual role in the soil microbial environment. In addition to functioning as a nitrogen source, Glu likely contributes to enhanced bacterial diversity by fulfilling microbial nutritional demands through amino acid metabolic pathways. However, direct experimental evidence supporting the role of Glu as an amino acid modulator of microbial diversity remains lacking. Future work will focus on disentangling the causal relationships among Glu, pH, and bacterial community structure.

Concurrently, we hypothesize that beyond serving as a nitrogen source, Glu may directly promote bacterial diversity by meeting microbial nutritional requirements through amino acid metabolic pathways. However, this study does not provide direct evidence supporting the role of Glu as an amino acid in modulating bacterial diversity. Further experimental investigations are planned to elucidate the causal relationships among Glu, pH, and bacterial diversity.

This study identified Acidobacteriota, Pseudomonadota, Chloroflexota, and Actinomycetota as the dominant rhizosphere bacterial phyla, aligning with prior research (Wang C. et al., 2018; Weng et al., 2023). Glu supplementation significantly increased the absolute abundance of Acidobacteriota and Pseudomonadota. Acidobacteriota, ubiquitous across diverse ecosystems, exhibit metagenomic-level functional traits linked to amino acid and carbohydrate metabolism, indirectly modulating plant growth. Additionally, they demonstrate enzymatic activity in degrading cellulose, dextran, starch, and peptidoglycan (Gonçalves et al., 2024). Soil pH is a critical regulator of Acidobacteriota dynamics, with their abundance inversely correlated with pH (Chen et al., 2024). The observed abundance increase may stem from Glu-induced soil acidification via hydrogen ion release during hydrolysis. Furthermore, Acidobacteriota’s strong association with nitrogen cycling suggests Glu-mediated nitrogen enrichment likely enhanced their competitiveness (Gonçalves et al., 2024). Pseudomonadota exhibited parallel abundance trends to Acidobacteriota, reflecting shared sensitivities to pH and nitrogen availability. Previous studies have established Pseudomonadota’s responsiveness to irrigation and nitrogen inputs, irrespective of nitrogen form (Zhang et al., 2014). Notably, absolute quantification revealed Glu-driven increases in total bacterial biomass across treatments, contrasting with the relative abundance trade-offs observed in compositional analyses.

Previous studies have demonstrated that nitrogen supplementation elevates soil inorganic nitrogen leaching, nitrification, nitrous oxide emissions, and denitrification (Lu et al., 2011), a pattern corroborated by this study. Notably, total bacterial abundance, trends in differentially abundant phyla, and the expression profiles of most nitrogen cycle genes peaked at the G5 treatment before declining. This implies that the benefits of Glu supplementation to soil systems are subject to diminishing returns, likely governed by a concentration-dependent threshold (Zhang et al., 2017). In this study, 5 mmol/L Glu appears to delineate this threshold, where maximal increases in nitrogen fixation-, nitrification-, and denitrification-associated gene abundances occurred. It should be noted that PICRUSt2 functional predictions depend on phylogenetic extrapolation from 16S rRNA data to annotated reference genomes (Douglas et al., 2020). In our analysis, 33% of ASVs were mapped to reference genomes with an NSTI value ≤ 0.2 (threshold: 2), while 75% of ASVs were mapped to those with an NSTI ≤ 0.5. These results suggest that a substantial proportion of the microbial community is phylogenetically closely related to sequenced taxa in reference databases. These methodological constraints must be explicitly acknowledged during ecological interpretation, particularly for nitrogen cycling processes given their enzymatic complexity, through either undetected functional contributions or distortion of predicted gene abundance ratios. Its accuracy exhibits reduced reliability in ecologically complex or poorly characterized ecosystems; consequently, metagenomic approaches remain the preferred methodology for detecting authentic gene abundance variations.

As an amino acid and nitrogen source, Glu can be efficiently utilized by plants; however, its cost about three–four times that of traditional nitrogen fertilizers (Table 3, data sourced from https://www.1688.com, with price fluctuations recorded as of 4/18/2025). Although less cost-effective, Glu offers advantages in water-soluble application and superior plant uptake efficiency compared to conventional nitrogen fertilizers, making it particularly suitable for rapid nitrogen supplementation during plant nitrogen deficiency.

**TABLE 3:** Prices of different types of nitrogen fertilizers.

	Glu	Ammonium bicarbonate	Ammonium sulfate	Ammonium chloride	Urea
Nitrogen content %	9	17	20	24	46
Price USD/ton	2,050	480	590	560	710

This study offers insights into rhizosphere microbial community dynamics following root application of Glu as an exogenous N/amino acid in Camellia species (or analogous crops) in agricultural production, while also providing guidance on optimal application concentrations.

Notably, this study has several limitations. First, the study revealed a significant decrease in soil pH after Glu application, suggesting that prolonged use of Glu may accelerate soil acidification. Co-application with quicklime and other alkaline matter or implementation in alkaline soil conditions (pH > 7.5) may constitute a preferable approach. Second, the 30-day pot experiment captured short-term microbial responses, which may reflect transient adaptation fluctuations, and further temporal variations cannot be excluded. Third, the study did not assess the effects of Glu on Camellia oil tree growth. Future field-based studies should investigate the long-term impacts of Glu on rhizosphere microorganisms and plant development, clarifying whether short-term microbial fluctuations persist and whether adverse effects on plants emerge. Such work could explore broader applications of Glu in agroforestry systems, establishing a robust theoretical foundation for developing precise and sustainable agricultural practices.

## 5 Conclusion

This study demonstrated that Glu supplementation significantly altered soil available nitrogen dynamics, including DON, ammonium NH_4_^+^-N, and NO_3_^–^-N. Absolute quantification revealed that escalating Glu concentrations enhanced the abundance of Acidobacteriota and Pseudomonadota, resulting in a unimodal trend of total bacterial abundance (initial increase followed by decline). The 5 mmol/L Glu treatment emerged as a critical threshold, beyond which bacterial abundance and nitrogen cycle-related gene expression in differential taxa declined. These findings collectively suggest that 5 mmol/L Glu represents a pivotal concentration influencing rhizosphere bacterial community dynamics in Camellia oil tree under drought stress.

## Data Availability

The raw sequence data reported in this paper have been deposited in the Genome Sequence Archive (Genomics, Proteomics & Bioinformatics 2021) in National Genomics Data Center (Nucleic Acids Res 2022), China National Center for Bioinformation/Beijing Institute of Genomics, Chinese Academy of Science (GSA: CRA024149) that are publicly accessible at https://ngdc.cncb.ac.cn/gsa.

## Footnotes

1
https://github.com/gastonstat/plspm

## References

[B1] Akter S. D’Ambra J. Ray P. (2011). “An evaluation of pls based complex models: The roles of power analysis, predictive relevance and gof index,” in *Proceedings of the AMCIS 2011 proceedings - all submissions*, Detroit, MI.

[B2] Aransiola S. A. Afolabi F. Joseph F. Maddela N. R. (2022). “Soil enzymes: Distribution, interactions, and influencing factors,” in *Agricultural biocatalysis*, eds Aransiola S. A. Afolabi F. (Singapore: Jenny Stanford Publishing).

[B3] Asgher M. Sehar Z. Rehaman A. Rashid S. Ahmed S. Per T. S. (2022). Exogenously-applied L-glutamic acid protects photosynthetic functions and enhances arsenic tolerance through increased nitrogen assimilation and antioxidant capacity in rice (*Oryza sativa* L.). *Environ. Pollut.* 301:119008. 10.1016/j.envpol.2022.119008 35189299

[B4] Berendsen R. L. Pieterse C. M. J. Bakker P. A. H. M. (2012). The rhizosphere microbiome and plant health. *Trends Plant Sci.* 17 478–486. 10.1016/j.tplants.2012.04.001 22564542

[B5] Bogati K. Walczak M. (2022). The impact of drought stress on soil microbial community, enzyme activities and plants. *Agronomy* 12:189. 10.3390/agronomy12010189

[B6] Bolan N. S. Hedley M. J. (2003). “Role of carbon, nitrogen, and sulfur cycles in soil acidification,” in *Handbook of soil acidity*, ed. Rengel Z. (Perth: University of Western Australia Perth).

[B7] Bolyen E. Rideout J. R. Dillon M. R. Bokulich N. A. Abnet C. C. Al-Ghalith G. A. (2019). Reproducible, interactive, scalable and extensible microbiome data science using QIIME 2. *Nat. Biotechnol.* 37 852–857. 10.1038/s41587-019-0209-9 31341288 PMC7015180

[B8] Callahan B. J. McMurdie P. J. Rosen M. J. Han A. W. Johnson A. J. A. Holmes S. P. (2016). DADA2: High-resolution sample inference from Illumina amplicon data. *Nat. Methods* 13 581–583. 10.1038/nmeth.3869 27214047 PMC4927377

[B9] Chen H. Li D. Zhao J. Xiao K. Wang K. (2018). Effects of nitrogen addition on activities of soil nitrogen acquisition enzymes:A meta-analysis. *Agricult. Ecosyst. Environ.* 252 126–131. 10.1016/j.agee.2017.09.032

[B10] Chen X. Wang Y. Wei H. Zhang J. (2024). Nitric acid rain decreases soil bacterial diversity and alters bacterial community structure in farmland soils. *Agronomy* 14:971. 10.3390/agronomy14050971

[B11] Chen Y. Wei T. Sha G. Zhu Q. Liu Z. Ren K. (2022). Soil enzyme activities of typical plant communities after vegetation restoration on the Loess Plateau, China. *Appl. Soil Ecol.* 170:104292. 10.1016/j.apsoil.2021.104292

[B12] Daniels M. B. Delaune P. Moore P. A. Jr. Mauromoustakos A. Chapman S. L. Langston J. M. (2001). Soil phosphorus variability in pastures: Implications for sampling and environmental management strategies. *J. Environ. Qual.* 30 2157–2165. 10.2134/jeq2001.2157 11790027

[B13] Dixon P. (2003). VEGAN, a package of R functions for community ecology. *J. Veg. Sci.* 14 927–930. 10.1111/j.1654-1103.2003.tb02228.x

[B14] Dong L. Berg B. Gu W. Wang Z. Sun T. (2022). Effects of different forms of nitrogen addition on microbial extracellular enzyme activity in temperate grassland soil. *Ecol. Proc.* 11:36. 10.1186/s13717-022-00380-2

[B15] Dong Y. Adingo S. Song X. Liu S. Hu Y. Zhang J. (2025). Characteristics and quantifications of soil acidification under different land uses and depths in northern subtropical China. *Soil Tillage Res.* 250:106527. 10.1016/j.still.2025.106527

[B16] Douglas G. M. Maffei V. J. Zaneveld J. R. Yurgel S. N. Brown J. R. Taylor C. M. (2020). PICRUSt2 for prediction of metagenome functions. *Nat. Biotechnol.* 38 685–688. 10.1038/s41587-020-0548-6 32483366 PMC7365738

[B17] Dray S. Dufour A.-B. (2007). The ade4 package: Implementing the duality diagram for ecologists. *J. Statist. Softw.* 22 1–20. 10.18637/jss.v022.i04

[B18] Fortunato S. Nigro D. Lasorella C. Marcotuli I. Gadaleta A. De Pinto M. C. (2023). The role of Glutamine Synthetase (GS) and Glutamate Synthase (GOGAT) in the improvement of nitrogen use efficiency in cereals. *Biomolecules* 13:1771. 10.3390/biom13121771 38136642 PMC10742212

[B19] Franzoni G. Cocetta G. Trivellini A. Garabello C. Contartese V. Ferrante A. (2022). Effect of exogenous application of salt stress and glutamic acid on lettuce (*Lactuca sativa* L.). *Sci. Horticult.* 299:111027. 10.1016/j.scienta.2022.111027

[B20] Gai Z. Liu L. Zhang J. Liu J. Cai L. (2020). Effects of exogenous α-oxoglutarate on proline accumulation, ammonium assimilation and photosynthesis of soybean seedling (*Glycine max*(L.) Merr.) exposed to cold stress. *Sci. Rep.* 10:17017. 10.1038/s41598-020-74094-w 33046814 PMC7550343

[B21] Gao X. Xiao Y. Deng L. Li Q. Wang C. Li B. (2019). Spatial variability of soil total nitrogen, phosphorus and potassium in renshou county of sichuan basin, China. *J. Integrat. Agricult.* 18 279–289. 10.1016/S2095-3119(18)62069-6

[B22] Goldblith S. A. Proctor B. E. (1950). Photometric determination of catalase activity. *J. Biol. Chem.* 187 705–709. 10.1016/S0021-9258(18)56216-5 14803454

[B23] Gonçalves O. S. Fernandes A. S. Tupy S. M. Ferreira T. G. Almeida L. N. Creevey C. J. (2024). Insights into plant interactions and the biogeochemical role of the globally widespread *Acidobacteriota phylum*. *Soil Biol. Biochem.* 192:109369. 10.1016/j.soilbio.2024.109369

[B24] Hao T. Liu X. Zhu Q. Zeng M. Chen X. Yang L. (2022). Quantifying drivers of soil acidification in three Chinese cropping systems. *Soil Tillage Res.* 215:105230. 10.1016/j.still.2021.105230

[B25] Jackman R. H. Black C. A. (1952). Phytase activity in soils. *Soil Sci.* 73 117–126. 10.1097/00010694-195202000-00004

[B26] Jiang S.-Q. Yu Y.-N. Gao R.-W. Wang H. Zhang J. Li R. (2019). High-throughput absolute quantification sequencing reveals the effect of different fertilizer applications on bacterial community in a tomato cultivated coastal saline soil. *Sci. Total Environ.* 687 601–609. 10.1016/j.scitotenv.2019.06.105 31220714

[B27] Jones D. L. Willett V. B. (2006). Experimental evaluation of methods to quantify dissolved organic nitrogen (DON) and dissolved organic carbon (DOC) in soil. *Soil Biol. Biochem.* 38 991–999. 10.1016/j.soilbio.2005.08.012

[B28] Jones D. L. Shannon D. Junvee-Fortune T. Farrar J. F. (2005). Plant capture of free amino acids is maximized under high soil amino acid concentrations. *Soil Biol. Biochem.* 37 179–181. 10.1016/j.soilbio.2004.07.021

[B29] Kan C.-C. Chung T.-Y. Wu H.-Y. Juo Y.-A. Hsieh M.-H. (2017). Exogenous glutamate rapidly induces the expression of genes involved in metabolism and defense responses in rice roots. *BMC Genom.* 18:186. 10.1186/s12864-017-3588-7 28212609 PMC5316172

[B30] Kandeler E. Gerber H. (1988). Short-term assay of soil urease activity using colorimetric determination of ammonium. *Biol. Fert. Soils* 6 68–72. 10.1007/BF00257924

[B31] Kim D.-R. Jeon C.-W. Cho G. Thomashow L. S. Weller D. M. Paik M.-J. (2021). Glutamic acid reshapes the plant microbiota to protect plants against pathogens. *Microbiome* 9:244. 10.1186/s40168-021-01186-8 34930485 PMC8691028

[B32] Kirk P. L. (2002). *Kjeldahl method for total nitrogen.* Washington, DC: ACS Publications, 10.1021/ac60038a038

[B33] Kou B. Hui K. Miao F. He Y. Qu C. Yuan Y. (2022). Differential responses of the properties of soil humic acid and fulvic acid to nitrogen addition in the North China Plain. *Environ. Res.* 214:113980. 10.1016/j.envres.2022.113980 35998702

[B34] Kumar L. Futschik E. M. (2007). Mfuzz: A software package for soft clustering of microarray data. *Bioinformation* 2 5–7. 10.6026/97320630002005 18084642 PMC2139991

[B35] Lee H. J. Lee J. H. Wi S. Jang Y. An S. Choi C. K. (2021). Exogenously applied glutamic acid confers improved yield through increased photosynthesis efficiency and antioxidant defense system under chilling stress condition in *Solanum lycopersicum* L. cv. dotaerang dia. *Sci. Horticult.* 277:109817. 10.1016/j.scienta.2020.109817

[B36] Li G. Li C. Wang Y. Fu K. Wei J. Zeng Y. (2024). Effects of exogenous L-glutamic acid on dry matter accumulation distribution, grain filling characteristis and quality formation under post-anthesis drought stress in wheat. *J. Plant Nutrit. Fertil.* 30 848–862. 10.11674/zwyf.2024001

[B37] Liao H.-S. Chung Y.-H. Hsieh M.-H. (2022). Glutamate: A multifunctional amino acid in plants. *Plant Sci.* 318:111238. 10.1016/j.plantsci.2022.111238 35351313

[B38] Liu Y. Wilson A. J. Han J. Hui A. O’Sullivan L. Huan T. (2023). Amino acid availability determines plant immune homeostasis in the rhizosphere microbiome. *mBio* 14:e03424-22. 10.1128/mbio.03424-22 36786577 PMC10127609

[B39] Lu M. Yang Y. Luo Y. Fang C. Zhou X. Chen J. (2011). Responses of ecosystem nitrogen cycle to nitrogen addition: A meta-analysis. *New Phytol.* 189 1040–1050. 10.1111/j.1469-8137.2010.03563.x 21138438

[B40] Maghini D. G. Dvorak M. Dahlen A. Roos M. Doyle B. Kuersten S. (2024). Quantifying bias introduced by sample collection in relative and absolute microbiome measurements. *Nat. Biotechnol.* 42 328–338. 10.1038/s41587-023-01754-3 37106038

[B41] National Technical Committee for Agricultural Meteorology Standardization, (2015). *Agricultural drought level.* Beijing: General Administration of Quality Supervision, Inspection and Quarantine of the People’s Republic of China, National Standardization Administration.

[B42] Props R. Kerckhof F.-M. Rubbens P. De Vrieze J. Hernandez Sanabria E. Waegeman W. (2017). Absolute quantification of microbial taxon abundances. *ISME J.* 11 584–587. 10.1038/ismej.2016.117 27612291 PMC5270559

[B43] Qiu X.-M. Sun Y.-Y. Ye X.-Y. Li Z.-G. (2020). Signaling role of glutamate in plants. *Front. Plant Sci.* 10:1743. 10.3389/fpls.2019.01743 32063909 PMC6999156

[B44] Tan X. (2023). Advances in the molecular breeding of *Camellia oleifera*. *J. Central South Univ. Forestry Technol.* 43 1–24. 10.14067/j.cnki.1673-923x.2023.01.001

[B45] Tkacz A. Hortala M. Poole P. S. (2018). Absolute quantitation of microbiota abundance in environmental samples. *Microbiome* 6:110. 10.1186/s40168-018-0491-7 29921326 PMC6009823

[B46] Wang C. Liu D. Bai E. (2018). Decreasing soil microbial diversity is associated with decreasing microbial biomass under nitrogen addition. *Soil Biol. Biochem.* 120 126–133. 10.1016/j.soilbio.2018.02.003

[B47] Wang F. Gao J. Yong J. W. H. Liu Y. Cao D. He X. (2020). Glutamate over-accumulation may serve as an endogenous indicator of tricarboxylic acid (TCA) cycle suppression under NH_4_^+^ nutrition in wheat (*Triticum aestivum* L.) seedlings. *Environ. Exp. Botany* 177:104130. 10.1016/j.envexpbot.2020.104130

[B48] Wang M. Ge A.-H. Ma X. Wang X. Xie Q. Wang L. (2024). Dynamic root microbiome sustains soybean productivity under unbalanced fertilization. *Nat. Commun.* 15:1668. 10.1038/s41467-024-45925-5 38395981 PMC10891064

[B49] Wang Q. Wang C. Yu W. Turak A. Chen D. Huang Y. (2018). Effects of nitrogen and phosphorus inputs on soil bacterial abundance, diversity, and community composition in Chinese fir plantations. *Front. Microbiol.* 9:1543. 10.3389/fmicb.2018.01543 30072961 PMC6060263

[B50] Weng X. Wang M. Sui X. Frey B. Liu Y. Zhang R. (2023). High ammonium addition changes the diversity and structure of bacterial communities in temperate wetland soils of Northeastern China. *Microorganisms* 11:2033. 10.3390/microorganisms11082033 37630593 PMC10459003

[B51] Wetzels M. Odekerken-Schröder G. van Oppen C. (2009). Using PLS path modeling for assessing hierarchical construct models: Guidelines and empirical illustration. *MIS Quar.* 33 177–195. 10.2307/20650284

[B52] Wickham H. (2016). *ggplot2, Use R!.* Cham: Springer International Publishing. 10.1007/978-3-319-24277-4

[B53] Yuan A. Kumar S. D. Wang H. Wang S. Impa S. Wang H. (2024). Dynamic interplay among soil nutrients, rhizosphere metabolites, and microbes shape drought and heat stress responses in summer maize. *Soil Biol. Biochem.* 191:109357. 10.1016/j.soilbio.2024.109357

[B54] Zhang J. Ai Z. Liang C. Wang G. Xue S. (2017). Response of soil microbial communities and nitrogen thresholds of *Bothriochloa ischaemum* to short-term nitrogen addition on the loess plateau. *Geoderma* 308 112–119. 10.1016/j.geoderma.2017.08.034

[B55] Zhang X. Wei H. Chen Q. Han X. (2014). The counteractive effects of nitrogen addition and watering on soil bacterial communities in a steppe ecosystem. *Soil Biol. Biochem.* 72 26–34. 10.1016/j.soilbio.2014.01.034

[B56] Zheng G. Liu C. Deng Z. Wei Z. Zhao Y. Qi H. (2021). Identifying the role of exogenous amino acids in catalyzing lignocellulosic biomass into humus during straw composting. *Bioresource Technol.* 340:125639. 10.1016/j.biortech.2021.125639 34315126

[B57] Zhou S. Yu B. Zhang Y. (2023). Global concurrent climate extremes exacerbated by anthropogenic climate change. *Sci. Adv.* 9:eabo1638. 10.1126/sciadv.abo1638 36897946 PMC10005174

