# Supplementary material for: Absolute quantification revealed that glutamate increased the abundance of the rhizosphere bacterial community in Camellia oil tree under drought stress
Source: Front Microbiol. 2025 Jun 13;16:1598000. doi: 10.3389/fmicb.2025.1598000 (PMC12202610; doi:10.3389/fmicb.2025.1598000)
Supplement: Supplementary file 2 [file Table_2.docx]

Table S1 Crossloading with different latent variables

|  | **Glu** | **Soil_nutrient** | **Soil_enzyme** | **Bacteria alpha diversity** |
| --- | --- | --- | --- | --- |
| **Glu** |  |  |  |  |
| 1 Glu | 1.00 | 0.88 | 0.73 | 0.89 |
| **Soil_nutrient** |  |  |  |  |
| 1 pH | -0.66 | -0.66 | -0.22 | -0.54 |
| 2 SOC | 0.52 | 0.76 | 0.46 | 0.49 |
| 3 TK | 0.59 | 0.80 | 0.45 | 0.34 |
| 4 AP | 0.79 | 0.93 | 0.48 | 0.53 |
| 5 DON | 0.91 | 0.87 | 0.56 | 0.76 |
| **Soil_enzyme** |  |  |  |  |
| 1 Phy | 0.70 | 0.58 | 0.88 | 0.65 |
| 2 BG | 0.48 | 0.28 | 0.75 | 0.61 |
| **Bacteria alpha**  **diversity** |  |  |  |  |
| 1 Shannon | 0.89 | 0.68 | 0.77 | 1.00 |

Table S2 Correlations between LVs

|  | Glu | Soil_nutrient | Soil_enzyme | Bacteria alpha diversity |
| --- | --- | --- | --- | --- |
| Glu | 1.00 | 0.88 | 0.73 | 0.89 |
| Soil_nutrient | 0.88 | 1.00 | 0.55 | 0.68 |
| Soil_enzyme | 0.73 | 0.55 | 1.00 | 0.77 |
| Bacteria alpha diversity | 0.89 | 0.68 | 0.77 | 1.00 |

Table S3 Manifest Variables and contribution values

| Relationships | Effect | | |
| --- | --- | --- | --- |
|  | Direct | Indirect | Total |
| Glu – Soil_nutrient | 0.88 | 0.00 | 0.88 |
| Glu –Soil_enzyme | 1.12 | -0.39 | 0.73 |
| Glu – Bacteria alpha diversity | 1.09 | -0.21 | 0.89 |
| Soil_nutrient –Soil_enzyme | -0.44 | 0.00 | -0.44 |
| Soil_nutrient – Bacteria alpha diversity | -0.38 | -0.08 | -0.46 |
| Soil_enzyme – Bacteria alpha diversity | 0.18 | 0.00 | 0.18 |
